# Long-term kidney outcomes in children after allogeneic hematopoietic stem cell transplantation assessed with estimated glomerular filtration rate equations, creatinine levels, and cystatin C levels

**DOI:** 10.1590/2175-8239-JBN-2021-0231en

**Published:** 2022-07-04

**Authors:** Aysha Gadashova, Seçil Conkar Tunçay, Gülcihan Özek, Gülden Hakverdi, Savaş Kansoy, Caner Kabasakal, Serap Aksoylar

**Affiliations:** 1 University Faculty of Medicine, Department of Pediatric Nephrology, İzmir, Turkey; 2 University Faculty of Medicine, Department of Pediatric Bone Marrow Transplantation, İzmir, Turkey; 3 University Faculty of Medicine, Department of Biostatistics and Medical Informatics, İzmir, Turkey

**Keywords:** Hematopoietic Stem Cell Transplantation, Transplantation, Homologous, Glomerular Filtration Rate, Renal Insufficiency, Chronic, Child, Cystatin C.

## Abstract

**Background and objective:**

With the widespread use of allogeneic hematopoietic stem cell transplantation (allo-HSCT), long-term complications have come to the fore. The aim of this study was to determine the prevalence and risk factors of chronic kidney disease (CKD) developing in the long term in patients who underwent allo-HSCT in childhood and also to investigate the superiority of eGFR formulas.

**Methods:**

The present study evaluated CKD in patients who underwent allo-HSCT. We analyzed the 94 children who received allo-HSCT at the Ege University in İzmir between August and November, 2019. The patients were evaluated at 2 years after transplantation. CKD was defined as a glomerular filtration rate (GFR) <90 mL/min/1.73 m^2^ using eGFR equations based on serum creatinine (SCr), cystatin C (CysC), and SCr plus CysC.

**Results:**

In our study, 9 (9.4%), according to Bedside Schwartz, 59 (76.6%), according to CKiD-eGFR-CysC, and 20 (26%) patients, according to CKiD-eGFR-SCr-CysC equations were identified with CKD. In cases identifies as CKD according to CysC, early development of acute kidney injury (AKI), post-transplant cytomegalovirus (CMV) reactivation and being >120 months during transplantation were found to be associated with the development of CKD.

**Conclusion:**

We may be delayed in detecting CKD by calculating SCr-based formulas in allo-HSCT cases, which is a patient group where early diagnosis and treatment of CKD is very important.

## Introduction

Allogeneic hematopoietic stem cell transplantation (allo-HSCT) is the curative treatment of many malignant and non-malignant congenital diseases, especially hematological malignancies in childhood. Although transplantation-related mortality has gradually decreased in recent years, especially with appropriate donor selection and supportive treatments, long-term complications are still an important issue^1^. Radiotherapy, calcineurin inhibitors (CNI), chemotherapy agents, hypertension, and graft-versus-host disease (GVHD) are known risk factors for chronic kidney disease (CKD) in patients undergoing HSCT^2^. CKD is a possible complication of HSCT and the most serious consequence is an end-stage renal disease (ESRD)^3^. Identifying risk factors, early diagnosis, and treatment of CKD before or after allo-HSCT are important for safe transplantation^4^.

Serum creatinine (SCr) value is the most commonly used marker for evaluating kidney function and calculating the estimated glomerular filtration rate (eGFR). However, the SCr is not an ideal biomarker because its serum level does not increase until there is a serious decrease in kidney function, as it is affected by many extrarenal factors. Therefore, serum cystatin C (CysC) has been used to evaluate kidney functions recently. CysC is a low molecular weight protein and highly correlated with eGFR. This relationship is independent of muscle mass, gender, body composition, or age^5^. The aim of this study was to determine the prevalence and risk factors of long-term CKD in children who underwent allo-HSCT and the ability of eGFR formulas in determining kidney functions.

## Methods

### Patient Selection

Between August and November 2019, 94 children over 24 months of age who underwent allo-HSCT at least 2 years before in the Pediatric Stem Cell Transplantation Center of the Faculty Medicine of the Ege University were included in the study. Ethics committee approval for this study was obtained from the Faculty of Medicine Medical Research Ethics Committee (Decision number: 19-10.IT/62). Patients with more than one HSCT, thyroid dysfunction, and corticosteroid use over 2 mg/kg/day were excluded from the study.

### Evaluation and Measurements

Demographic information before allo-HSCT, primary diagnosis and treatments, preparation regimens used during HSCT, presence of total body irradiation (TBI), donor characteristics, stem cell sources, early post-transplant complications such as veno-occlusive disease (VOD), GVHD, cytomegalovirus (CMV), BK, and adenovirus infections, hemorrhagic cystitis and urinary tract infections (UTI), development of sepsis, intensive care unit (ICU) admissions, GVHD prophylactic treatments, use of CNI and nephrotoxic antimicrobial drugs, acute kidney injury (AKI) after transplantation and dialysis requirements of the patients were retrospectively obtained from hospital files. During the study, the patients’ kidney function tests, complete urine analysis, urine protein/creatinine and albumin/creatinine ratios, urine beta-2 microglobulin levels, and the presence of hematuria were evaluated, and eGFR was calculated according to SCr and CysC. GFR values were interpreted according to the Kidney Disease Improving Global Outcomes (KDIGO) 2012 classification. Pediatric RIFLE criteria were used for the diagnosis of AKI^6^. For the staging of CKD, KDIGO 2012 practice guidelines were referred and CKD was defined as eGFR<90 mL/min/1.73 m^2^. Bedside Schwartz^7,8^, CKiD-eGFR-CysC^9^, and CKiD-eGFR-SCr-CysC^10^ formulas were used for eGFR calculation based on SCr, CysC, and SCr plus CysC, respectively. CysC was studied by the immunonephelometric method with the Siemens-BN II nephelometer device. Roche Diagnostics kits and modular cobas® automated analyzers were used for other biochemical parameters. Systolic (SBP) and diastolic blood pressures (DBP) were measured with an oscillometric method by the Omron automatic device. Blood pressure measurements were done after 5 minutes of rest. Hypertension was defined as SBP and/or DBP z-score greater than 1.65 SDS (95th percentile) for sex, age, and height^11^. Microalbuminuria is defined as albumin excretion >0.3 mg/mg creatinine^12^.

### Transplant Procedure

The preparatory regimen was myeloablative in 82 cases, non-myeloablative in 6 cases, and a reduced toxicity regimen in 3 cases. In the preparatory regimen, different combinations of cyclophosphamide, fludarabine, melphalan, etoposide, busulfan, anti-thymocyte globulin (ATG), thiotepa, threosulfan, alemtuzumab, and TBI (fractionated 2x2 Gy, 3 days) were administered to the patients. As a GVHD prophylaxis, cyclosporine (CsA) 3 mg/kg/day, CsA 3 mg/kg/day plus short-term methotrexate 10 mg/m^2^/day (+1, 3, 6^th^ days), CsA 3 mg/kg/day plus mycophenolate mofetil 15 mg/kg TID, CsA 3 mg/kg/day plus corticosteroid 1 mg/kg/day, and prophylactic acyclovir, fluconazole, and trimethoprim-sulfamethoxazole were given to 91 patients. Four patients did not receive GVHD prophylaxis.

### Statistical Analysis

Continuous variables were summarized with mean ± SD, median (min. max.). Categorical variables were described as frequencies and percentage and compared with Chi-square test. IBM SPSS Statistics 25.0 (IBM SPSS Statistics for Windows, Version 25.0. Armonk, NY: IBM Corp.) software was used for statistical analysis. Binary logistic regression analysis was performed to determine CKD risk factors. The statistical significance level was determined as p <0.05 in all analyses of the study.

## Results

The mean age of the patients included in the study was 152.6 ± 67 months [61.7% male (58 months) and 38.2% female (36 months)], the age at diagnosis was 49.43 ± 51.75 months, and the mean age when HSCT was performed was 91.18 ± 59.39 months. The period after transplantation was 62.53 ± 34.95 months. The clinical and demographic characteristics of the patients and information about the HSCT procedure are shown in Table 1.

**Table 1:** The clinical and demographic characteristics of the patients and information about the HSCT procedure

Patients		n (%)
Sex	Female	36 (38.2)
	Male	58 (61.7)
Diagnosis	Hematological malignancies	35 (37.3)
	Bone marrow failure	6 (6.4)
	Hemoglobinopathies	18 (19.1)
	Primary Immunodeficiencies	24 (25.5)
	Histiocytosis (HLH + LCH)	6 (6.4)
	Osteopetrosis	5 (5.3)
Stem cell source	Peripheral stem cell	46 (48.9)
	Bone marrow	45 (47.8)
	Cord blood	3 (3.2)
Donor	Matched sibling donor	33 (35.12)
	Matched related donor	10 (10.63)
	Matched unrelated donor (10/10 and 9/10)	35 (37.23)
	Haploidentical donor	16 (17.02)
Preparation regimen	Myeloablative	81 (86.2)
	Non-myeloablative	6 (6.3)
	Reduced toxicity	3 (3.2)
	Regime not given	4 (4.2)
TBI	Yes	18 (19.1)
GVHD prophylaxis	Yes	91 (96.8)
	CSA	11 (11.7)
	CSA + methotrexate	61 (64.9)
	CSA + mycophenolate mofetil	13 (13.8)
	CSA + steroid	5 (5.3)
	CSA + corticosteroid	1 (1.1)
	No	3 (3.2)

AML: acute myeloid leukemia, ALL: acute lymphoblastic leukemia, CML: chronic myeloid leukemia, HLH: hemophagocytic lymphohistiocytosis, LCH: Langerhans cell histiocytosis, GVHD: graft versus host disease, TBI: total body irradiation. CSA: cyclosporin.

GVHD developed in 49 (51%) cases in the early post-transplant period (28 acute and 22 chronic GVHD). CMV infection developed in 43 (44.8%) cases, adenovirus in 3 (3.1%), BK virus-associated hemorrhagic cystitis in 16 (16.7%), and UTI in 23 (24%). AKI developed in 33 (34.4%) cases, but no patients required dialysis. VOD developed in 17 cases (17.7%), sepsis in 62 cases (64.6%), and 12 cases (12.5%) were hospitalized in Pediatric ICU. Amikacin was used in 65 (67.7%) cases, vancomycin in 20 (20.8%), and amphotericin B in 20 (20.8%) (Table 2).

**Table 2:** Early complications after HSCT

Complications	n	%
GVHD	49	51
Acute	28	29.7
Chronic	21	23.4
CMV	43	44.8
BK virus-associated hemorrhagic cystitis	16	16.7
Adenovirus	3	3.124
UTI	23	24.5
AKI		
Yes	33	34.4
No	61	63.5
Sepsis	62	64.6
Intensive Care Hospitalization	12	12.5
Amikacin use	65	67.7
Vancomycin use	20	20.8
Amphotericin B use	20	20.8
Ganciclovir use	43	44.8
VOD	17	17.7

GVHD: graft versus host disease, CMV: cytomegalovirus, UTI: urinary tract infection, AKI: acute kidney injury, VOD: veno-occlusive disease.

CysC was measured in 77 of 94 cases included in the study. GFR was calculated according to three different formulas (Bedside Schwartz, eGFR-CysC, eGFR-SCr-CysC). When the values were evaluated based on the KDIGO 2012 clinical practice guideline, it was seen that GFR was ≥90 mL/min/1.73 m^2^ in 85 (90.4%) and 60 - 89 mL/min/1.73 m^2^ in 9 (9.4%) patients according to Bedside Schwartz formula. Stage 3, 4, and 5 CKD were not detected. According to eGFR-CysC, GFR was ≥90 mL/min/1.73 m^2^ in 18 (23.3%), 53 (68.8%) were stage 2, and 6 (7.7%) cases were stage 3a. Stages 4 and 5 were not detected. According to eGFR-SCr-CysC formula, GFR was ≥90 mL/min/1.73 m^2^ in 57 (74%) cases, 19 (24.7%) cases were stage 2, and 1 (1.3%) patient was stage 3a. Eventually, when CKD was described as GFR<90 mL/min/1.73 m^2^, the frequency of CKD was 9.4% according to Bedside Schwartz, 76.6% according to eGFR-CysC, and 25.9% according to eGFR-SCr-CysC formulas in our study (Table 3).

**Table 3:** CKD prevalence according to Bedside Schwartz eGFR, eGFR-CysC, and eGFR-SCr-cysC formulas

Stage	Stage 1 ≥90 n (%)	Stage 2 60-89 n (%)	Stage 3a 45-59 n (%)	Total
eGFR (mL/min/1.73 m^2^)				
Bedside Schwartz	85 (90.4%)	9 (9.4%)	-	94
eGFR-CysC	18 (23.3%)	53 (68.8%)	6 (7.7%)	77
eGFR-SCr-CysC	57 (74%)	19 (24.7%)	1 (1.3%)	77

There was no statistically significant correlation between CKD and clinical factors such as acute and chronic GVHD, preparation regimen, TBI, VOD, CNI use, BK virus-associated hemorrhagic cystitis, UTI, donor compliance, development of sepsis, ICU admission, and nephrotoxic antimicrobial medication according to Bedside Schwartz, eGFR-CysC, and eGFR-SCr-CysC equations (p>0.05). There was no significant relationship between AKI and CMV reactivation and Bedside Schwartz and eGFR-SCr-CysC formulas (p=0.907 and p=0.325, respectively). According to eGFR-CysC, CMV reactivation caused a 4.2-fold increase in CKD development (OR=4.2, p=0.041). When calculated with the eGFR-CysC formula, the risk of CKD was higher in cases who underwent HSCT at more than 120 months of age (OR=3.359, p=0.03) (Table 4).

**Table 4:** Univariate logistic regression analysis of risk factors of CKD (eGFR-CysC)

Characteristics (n)	OR (95% CI)	*P*-value
Age at HSCT	3.359 (1.127-10.018)	0.03
Malignant diseases	1.070 (0.362-3.166)	0.902
Acute kidney injury	0.159 (0.033.-0.753)	0.02
Acute GVHD	0.178 (0.017-1.862)	0.150
Chronic GVHD	0.500(0.033-7.541)	0.617
Total body irradiation	2.450 (0.743-8.076)	0.141
Veno-occlusive disease	1.111 (0.266-4.635)	0.885
Calcineurin inhibitor in months	0.347(0.110-1.098)	0.072
BK virus	0.230 (0.028-1.908)	0.174
Intensive care hospitalization	1.486(0.347-6.458)	0.597
Sepsis development	1.661(0.523-5.280)	0.390
CMV reactivation	4.2 (1.059-16.259)	0.041
Ganciclovir use	0.422 (0.140-1.275)	0.126
Vancomycin use	1.247(0.343-4.529)	0.738
Amphotericin B use	1.587(0.425-5.933)	0.492
Amikacin use	2.763 (0.717-10.651)	0.140

Items with statistical significance (P<.05) are shown in bold type. GVHD: graft versus host disease, CMV: cytomegalovirus.

With regards to proteinuria, hematuria, and hypertension for long-term renal impairment, protein/creatinine in spot urine was >0.21 g/day in 9 (9.4%) cases, and microalbuminuria was found in 18 (18.8%) patients. The β2-microglobulin, examined in terms of tubular functions, was found to be >300 mcg/L in 9 (9.4%) cases. Hematuria was observed in 13 (13.5%) cases. Sixty-eight (72.3%) cases were normotensive, 22 (23.4%) cases were pre-hypertensive, and 4 (4.3%) cases were hypertensive.

## Discussion

Although allo-HSCT is used with increasing frequency in the treatment of many diseases, concerns regarding long-term complications remain. One of the long-term complications of allo-HSCT is CKD. The development of CKD in allo-HSCT recipients has important medical and economic consequences, such as ESRD and cardiovascular morbidity, and may decrease the quality of life. Although early kidney complications after HSCT are well defined, long-term effects are still less known^13,14^.

In this study, unlike other studies, long-term kidney functions and risk factors for kidney disease were evaluated with different eGFR formulas using serum CysC and SCr in patients without recurrence at least 2 years after allo-HSCT recipients. The eGFR was evaluated according to Bedside Schwartz, eGFR-CysC, and eGFR-SCr-CysC formulas. Because SCr is affected by malnutrition or tubular secretion, in this study we calculated eGFR with equations based on CysC as well as SCr. Chemotherapeutics and radiotherapy used in the preparatory regimen, nephrotoxic antimicrobial drugs, AKI, and GVHD have been shown to be among the CKD risk factors after HSCT^15^. In the study of Abboud et al., renal dysfunction was found in 7% of children with HSCT, and the development of CKD was associated with TBI and chronic GVHD^16^. Administration of TBI during preparation and chronic GVHD is one of the main causes of late kidney disease^17^. In addition, it has been shown that the age of transplant over 84 months is a risk for low eGFR in the long term after HSCT in all patients^18^. In the study performed by Sakellari et al., the incidence of CKD was 20.4%. Besides, chronic GVHD, unrelated donor transplant, post-transplant AKI, and advanced age have been identified as risk factors for CKD^19^. In our study, in cases evaluated as CKD according to all three formulas, CKD development was not associated with TBI, BK virus, sepsis, VOD, donor type, and chronic GVHD. However, in patients identified as CKD according to the eGFR-CysC equation, transplantation age above 120 months, CMV infection, and AKI were found to be associated with CKD. Our study emphasizes the importance of CysC in HSCT cases since risk factors are only associated with CKD identified by the eGFR-CysC formula. Muto et al. revealed the relationship between CysC and CNI use. A strong inverse correlation was also noted between CysC and eGFR. All this supports that CysC may be a useful clinical marker to define the risk of CKD in allo-HSCT recipients^20^. The sooner CKD is diagnosed, the better progress to ESRD can be slowed by treatment and precautions.

Different results have been obtained in different publications investigating the frequency of CKD in allo-HSCT cases. In the study of Abboud et al., kidney dysfunction was found in 7%^16^ and in the study by Hingorani et al., the prevalence of CKD was found to be 23% after HSCT^21^. Y. Ukeba-Terashita et al. and Sakellari et al. reported CKD incidences of 21.7% and 20.4%, respectively^18,19^. In our study, different CKD prevalence rates were determined with different eGFR formulas. CKD prevalence was 9.3% according to Bedside Shwartz, 76.6% according to eGFR-CysC, and 25% according to eGFR-SCr-CysC equations. This suggests that Bedside Shwartz formula, based on SCr, which we use most frequently in daily practice, may mislead us in determining CKD. On the other hand, detection of CKD could be delayed by calculating eGFR based on SCr in HSCT cases, which is a patient group where early diagnosis and treatment of CKD is very important.

The use of SCr-based formulas to estimate GFR is potentially problematic. Creatinine is excreted through glomerular filtration and tubular secretion. SCr concentration may remain normal even if the GFR has dropped to 50% of the normal range. Conversely, CysC is expressed by all cells and is excreted only through glomerular filtration. Moreover, this substance is not secreted but instead is reabsorbed and catabolized by tubular epithelial cells, thus preventing return to blood. CysC offers superior diagnostic sensitivity for detection of slightly impaired GFR compared to SCr^21^. Hazar et al. recently reported that CysC-based GFR could be much closer to the currently accepted 99mTc-DTPA-based GFR method^23^. In this study, a strong correlation was found between CysC level and eGFR. The serum CysC level is therefore useful in predicting renal function in HSCT patients. In addition, CysC can be measured without urine collection, providing a great advantage for easy monitoring of renal function in pre-clinical settings^24^.

SCr is the most commonly used marker for the assessment of kidney function. CysC has been proposed as an alternative marker of kidney function for the eGFR, which seems to be more accurate than SCr. eGFR is considered the best overall index of renal function, allowing the early recognition of kidney dysfunction as well as the accurate dosing of immunosuppressive drugs. The strong dependence of SCr on age, gender, nutritional status, and lean muscle mass must be emphasized. This limitation is particularly important in patients in whom SCr could be misleadingly low due to muscle loss or hyperfiltration. Thus, SCr can only be used as a crude indicator of significantly impaired kidney function, is not an ideal marker for early recognition of acute kidney damage, and also does not reflect real-time eGFR changes. Therefore, in allo-HSCT recipients, creatinine-based equations have equivocal results in estimating GFR. CysC is a marker of kidney function that has better diagnostic sensitivity than SCr for the detection of mild to moderate impairments of GFR. It has been reported to be less affected by age, gender, and body composition^25^. Recent studies demonstrated that the CysC-based formula had a better predictive performance for eGFR than the SCr-based formula.

In this study, the risk of CKD was observed to be quite high in children who underwent allo-HSCT for different reasons (eGFR-CysC of 76.6%). Similar to the literature, we found CMV infection, transplant age greater than 120 months, and previous AKI as risk factors for CKD. Another important finding of our study was that the frequency of CKD varied depending on eGFR formulas. Since CKD is often asymptomatic in the early stages, early detection is important to prevent or slow down the progression to ESRD. In this sense, eGFR-CysC formula may be more effective than SCr in defining CKD after allo-HSCT. CysC resulted in better results among eGFR pediatric formulas in allo-HSCT patients. CKiD-eGFR-ScCr-CysC is currently considered the best predictive GFR equation for assessment of CKD progression in the pediatric age range. The new FAS (full age spectrum) equation is based on normalized serum creatinine (SCr/Q), where Q is the median SCr from healthy populations to account for age and sex^26^. We did not use the FAS formula since our study consisted of only pediatric patients.

The limitations of our study include the cross-sectional design, the lack of relationship between baseline eGFR and CKD, the inhomogeneity of patient groups, follow-up periods, preparatory regimens, age at the time of transplantation, donor type, and primary diagnoses.

In conclusion, CysC measurement is superior to SCr measurement for the assessment of kidney dysfunction and may provide a useful tool to identify the risk of CKD in allo-HSCT recipients. eGFR-CysC can be considered the most appropriate eGFR assessment method in pediatric transplants.
