# Erratum: Long-term kidney outcomes in children after allogeneic hematopoietic stem cell transplantation assessed with estimated glomerular filtration rate equations, creatinine levels, and cystatin C levels

**DOI:** 10.1590/2175-8239-JBN-2021-0231eren

**Published:** 2025-04-25

In the article “Long-term kidney outcomes in children after allogeneic hematopoietic stem cell transplantation assessed with estimated glomerular filtration rate equations, creatinine levels, and cystatin C levels”, with DOI code number:related-article https://doi.org/10.1590/2175-8239-JBN-2021-0231en, published in the journal Brazilian Journal of Nephrology, 45(1):60-66, 2023, in the page 66 the acknowledgments were missing:

