# Supplementary material for: Long-term kidney outcomes in children after allogeneic hematopoietic stem cell transplantation assessed with estimated glomerular filtration rate equations, creatinine levels, and cystatin C levels
Source: J Bras Nefrol. 2022 Jul 4;45(1):60–6. doi: 10.1590/2175-8239-JBN-2021-0231en (PMC10139723; doi:10.1590/2175-8239-JBN-2021-0231en)
Supplement: Translation

# Translation: Desfechos renais a longo prazo em crianças após transplante alogênico de células-tronco hematopoiéticas avaliados com equações de taxa de filtração glomerular estimada, níveis de creatinina e níveis de cistatina C

**DOI:** 10.1590/2175-8239-JBN-2021-0231pt

**Authors:** Aysha Gadashova, Seçil Conkar Tunçay, Gülcihan Özek, Gülden Hakverdi, Savaş Kansoy, Caner Kabasakal, Serap Aksoylar

**Affiliations:** 1 University Faculty of Medicine, Departamento de Nefrologia Pediátrica, İzmir, Turquia; 2 University Faculty of Medicine, Departamento de Transplante Pediátrico de Medula Óssea, İzmir, Turquia; 3 University Faculty of Medicine, Departamento de Bioestatística e Informática Médica, İzmir, Turquia

**Keywords:** Transplante de Células-Tronco Hematopoéticas, Transplante Homólogo, Taxa de Filtração Glomerular, Insuficiência Renal Crônica, Criança, Cistatina C.

## Abstract

**Antecedentes e objetivo:**

Com o uso generalizado do transplante alogênico de células-tronco hematopoiéticas (TCTH-alo), as complicações a longo prazo tornaram-se evidentes. O objetivo deste estudo foi determinar a prevalência e os fatores de risco do desenvolvimento de doença renal crônica (DRC) a longo prazo em pacientes submetidos a TCTH-alo na infância, e também investigar a superioridade das fórmulas de TFGe.

**Métodos:**

O presente estudo avaliou a DRC em pacientes que foram submetidos ao TCTH-alo. Analisamos as 94 crianças que receberam TCTH-alo na Universidade Ege em İzmir entre Agosto e Novembro de 2019. Os pacientes foram avaliados aos 2 anos após o transplante. A DRC foi definida como uma taxa de filtração glomerular (TFG) <90 mL/min/1,73 m^2^ usando equações de TFGe baseadas em creatinina sérica (CrS), cistatina C (CisC), e CrS mais CisC.

**Resultados:**

Em nosso estudo, 9 pacientes (9,4%), de acordo com a equação de Schwartz (à beira do leito), 59 (76,6%), de acordo com a equação DRC-TFGe-CisC, e 20 (26%) pacientes, de acordo com a equação DRC-TFGe-CrS-CisC, foram classificados com DRC. Quando a TFG é avaliada pela CisC, verificamos que o desenvolvimento precoce de lesão renal aguda (LRA), a reativação do citomegalovírus (CMV) pós-transplante e ter >120 meses durante o transplante foram associados ao desenvolvimento de DRC.

**Conclusão:**

Pode haver atraso na detecção da DRC quando usamos fórmulas baseadas em CrS em casos de TCTH-alo, que é um grupo de pacientes onde o diagnóstico e tratamento precoces da DRC são muito importantes.

## Introdução

O transplante alogênico de células-tronco hematopoiéticas (TCTH-alo) é o tratamento curativo de muitas doenças congênitas malignas e não malignas, especialmente neoplasias hematológicas na infância. Embora a mortalidade relacionada a transplantes tenha diminuído gradualmente nos últimos anos, especialmente com a seleção apropriada de doadores e tratamentos de suporte, as complicações a longo prazo ainda são uma questão importante^1^. Radioterapia, inibidores de calcineurina (ICN), agentes quimioterápicos, hipertensão e doença do enxerto contra hospedeiro (DECH) são fatores de risco conhecidos para doença renal crônica (DRC) em pacientes submetidos ao TCTH^2^. A DRC é uma possível complicação do TCTH e a consequência mais grave é uma doença renal em estágio terminal (DRET)^3^. A identificação dos fatores de risco, o diagnóstico precoce e o tratamento da DRC antes ou após o TCTH-alo são importantes para um transplante seguro^4^.

O valor da creatinina sérica (CrS) é o marcador mais comumente utilizado para avaliar a função renal e calcular a taxa de filtração glomerular estimada (TFGe). No entanto, a CrS não é um biomarcador ideal porque seu nível sérico não aumenta até que haja uma grave diminuição na função renal, uma vez que é afetada por muitos fatores extrarrenais. Portanto, a cistatina C (CisC) sérica tem sido utilizada recentemente para avaliar as funções renais. A CisC é uma proteína de baixo peso molecular e altamente correlacionada com a TFGe. Esta relação é independente de massa muscular, sexo, composição corporal, ou idade^5^. O objetivo deste estudo foi determinar a prevalência e os fatores de risco de DRC a longo prazo em crianças submetidas a TCTH-alo e a capacidade das fórmulas de TFGe na determinação das funções renais.

## Métodos

### Seleção de Pacientes

Entre Agosto e Novembro de 2019, foram incluídas no estudo 94 crianças com mais de 24 meses de idade submetidas a TCTH-alo pelo menos 2 anos antes, no Centro de Transplante Pediátrico de Células-Tronco da Faculdade de Medicina da Universidade de Ege. A aprovação do comitê de ética para este estudo foi obtida junto ao Comitê de Ética em Pesquisa Médica da Faculdade de Medicina (Número da decisão: 19-10.IT/62). Foram excluídos do estudo os pacientes com mais de um TCTH, disfunção tireoidiana e uso de corticosteroides acima de 2 mg/kg/dia.

### Avaliação e Medições

Informações demográficas antes do TCTH-alo, diagnóstico e tratamentos primários, regimes de preparação utilizados durante o TCTH, presença de irradiação corporal total (ICT), características do doador, fontes de células-tronco, complicações precoces pós-transplante tais como doença veno-oclusiva (DVO), DECH, citomegalovírus (CMV), BK, e infecções por adenovírus, cistite hemorrágica e infecções do trato urinário (ITU), desenvolvimento de sepse, internações em unidade de terapia intensiva (UTI), tratamentos profiláticos da DECH, uso de ICN e drogas antimicrobianas nefrotóxicas, lesão renal aguda (LRA) após o transplante e requisitos de diálise dos pacientes foram obtidos retrospectivamente dos arquivos hospitalares. Durante o estudo, foram avaliados os testes de função renal, a análise completa da urina, as relações proteína/creatinina e albumina/creatinina urinárias, os níveis de beta-2 microglobulina na urina e a presença de hematúria dos pacientes, e a TFGe foi calculada de acordo com CrS e CisC. Os valores da TFG foram interpretados de acordo com a classificação do KDIGO (*Kidney Disease Improving Global Outcomes*) de 2012. Os critérios RIFLE pediátricos foram utilizados para o diagnóstico de LRA^6^. Para os estágios da DRC, foram referidas as diretrizes práticas do KDIGO 2012 e a DRC foi definida como TFGe <90 mL/min/1,73 m^2^. As fórmulas de Schwartz (à beira do leito)^7,8^, DRC-TFGe-CisC^9^, e DRC-TFGe-CrS-CisC^10^ foram usadas para o cálculo da TFGe com base em CrS, CisC, e CrS mais CisC, respectivamente. A CisC foi estudada pelo método de imunonefelometria com o dispositivo nefelômetro Siemens-BNII. Os kits *Roche Diagnostics* e os analisadores automatizados modulares cobas® foram utilizados para outros parâmetros bioquímicos. As pressões arteriais sistólica (PAS) e diastólica (PAD) foram medidas com um método oscilométrico pelo dispositivo automático Omron. As medições de pressão arterial foram feitas após 5 minutos de repouso. A hipertensão foi definida como PAS e/ou PAD expressos em escore z maior que 1,65 SDS (percentil 95) para sexo, idade e altura^11^. A microalbuminúria é definida como excreção de albumina >0,3 mg/mg de creatinina^12^.

### Procedimento de Transplante

O regime preparatório foi mieloablativo em 82 casos, não-mieloablativo em 6 casos, e um regime de toxicidade reduzida em 3 casos. No regime preparatório, foram administradas aos pacientes diferentes combinações de ciclofosfamida, fludarabina, melfalano, etoposido, bussulfano, globulina antitimócito (ATG), tiotepa, treossulfan, alemtuzumab e ICT (fracionado 2x2 Gy, 3 dias). Como profilaxia da DECH foram administrados ciclosporina (CsA) 3 mg/kg/dia, CsA 3 mg/kg/dia mais metotrexato de curto prazo 10 mg/m^2^/dia (+1º, 3º, 6º dias), CsA 3 mg/kg/dia mais micofenolato de mofetila 15 mg/kg TID, CsA 3 mg/kg/dia mais corticosteroide 1 mg/kg/dia, e aciclovir profilático, fluconazol e sulfametoxazol-trimetoprima a 91 pacientes. Quatro pacientes não receberam profilaxia da DECH.

### Análise Estatística

As variáveis contínuas foram resumidas com média ± DP, mediana (mín. máx.). As variáveis categóricas foram descritas como frequências e percentual e comparadas com o teste Qui-quadrado. O software IBM SPSS Statistics 25.0 (IBM SPSS Statistics para Windows, Versão 25.0. Armonk, NY: IBM Corp.) foi usado para análise estatística. A análise de regressão logística binária foi realizada para determinar os fatores de risco de DRC. O nível de significância estatística foi determinado como p <0,05 em todas as análises do estudo.

## Resultados

A idade média dos pacientes incluídos no estudo foi de 152,6 ± 67 meses [61,7% homens (58 meses) e 38,2% mulheres (36 meses)], a idade no momento do diagnóstico foi de 49,43 ± 51,75 meses, e a idade média quando o TCTH foi realizado foi de 91,18 ± 59,39 meses. O período após o transplante foi de 62,53 ± 34,95 meses. As características clínicas e demográficas dos pacientes e as informações sobre o procedimento de TCTH são apresentadas na Tabela 1.

**Tabela 1:** Características clínicas e demográficas dos pacientes e informações sobre o procedimento de TCTH

Pacientes		n (%)
Sexo	Feminino	36 (38,2)
	Masculino	58 (61,7)
Diagnóstico	Neoplasias hematológicas	35 (37,3)
	Insuficiência da medula óssea	6 (6,4)
	Hemoglobinopatias	18 (19,1)
	Imunodeficiências primárias	24 (25,5)
	Histiocitose (LHH + HCL)	6 (6,4)
	Osteopetrose	5 (5,3)
Fonte de células-tronco	Célula-tronco periférica	46 (48,9)
	Medula óssea	45 (47,8)
	Sangue de cordão umbilical	3 (3,2)
Doador	Doador irmão compatível	33 (35,12)
	Doador relacionado compatível	10 (10,63)
	Doador não relacionado compatível (10/10 e 9/10)	35 (37,23)
	Doador haploidêntico	16 (17,02)
Regime de preparação	Mieloablativo	81 (86,2)
	Não-mieloablativo	6 (6,3)
	Toxicidade reduzida	3 (3,2)
	Regime não fornecido	4 (4,2)
ICT	Sim	18 (19,1)
Profilaxia da DECH	Sim	91 (96,8)
	CsA	11 (11,7)
	CsA + metotrexato	61 (64,9)
	CsA + micofenolato de mofetila	13 (13,8)
	CsA + esteroide	5 (5,3)
	CsA + corticosteroide	1 (1,1)
	Não	3 (3,2)

LMA: leucemia mieloide aguda, LLA: leucemia linfoblástica aguda, LMC: leucemia mieloide crônica, HLH: linfohistiocitose hemofagocítica, LCH: histiocitose de células de Langerhans, DECH: doença do enxerto contra hospedeiro, ICT: irradiação corporal total, CsA: ciclosporina.

A DECH desenvolveu-se em 49 (51%) casos no período inicial pós-transplante (28 casos agudos e 22 casos crônicos de DECH). A infecção por CMV se desenvolveu em 43 (44,8%) casos, adenovírus em 3 (3,1%), cistite hemorrágica associada ao vírus BK em 16 (16,7%), e ITU em 23 (24%). A LRA desenvolveu-se em 33 (34,4%) casos, mas nenhum paciente necessitou de diálise. Houve o desenvolvimento de DVO em 17 casos (17,7%), sepse em 62 casos (64,6%), e 12 casos (12,5%) foram hospitalizados em UTI Pediátrica. A amicacina foi utilizada em 65 (67,7%) casos, vancomicina em 20 (20,8%) e anfotericina B em 20 (20,8%). (Tabela 2).

**Tabela 2:** Complicações precoces após o TCTH

Complicações	n	%
DECH	49	51
Aguda	28	29,7
Crônica	21	23,4
CMV	43	44,8
Cistite hemorrágica associada ao vírus BK	16	16,7
Adenovírus	3	3,124
ITU	23	24,5
LRA		
Sim	33	34,4
Não	61	63,5
Sepse	62	64,6
Hospitalização em Terapia Intensiva	12	12,5
Uso de amicacina	65	67,7
Uso de vancomicina	20	20,8
Uso de anfotericina B	20	20,8
Uso de ganciclovir	43	44,8
DVO	17	17,7

DECH: doença do enxerto contra hospedeiro, CMV: citomegalovírus, ITU: infecção do trato urinário, LRA: lesão renal aguda, DVO: doença veno-oclusiva.

A CisC foi medida em 77 dos 94 casos incluídos no estudo. A TFG foi calculada de acordo com três fórmulas diferentes, (Schwartz (à beira do leito), TFGe-CisC, TFGe-CrS-CisC). Quando os valores foram avaliados com base na diretriz de prática clínica do KDIGO 2012, observou-se que a TFG foi ≥90 mL/min/1,73 m^2^ em 85 (90,4%) e 60 - 89 mL/min/1,73 m^2^ em 9 (9,4%) pacientes de acordo com a fórmula de Schwartz (à beira do leito). Não foram detectados os estágios 3, 4, e 5 da DRC. De acordo com a fórmula de TFGe-CisC, a TFG foi ≥90 mL/min/1,73 m^2^ em 18 casos (23,3%), 53 (68,8%) foram estágio 2, e 6 (7,7%) casos foram estágio 3a. Os estágios 4 e 5 não foram detectados. De acordo com a fórmula de TFGe-CrS-CisC, a TFG foi ≥90 mL/min/1,73 m^2^ em 57 (74%) casos, 19 (24,7%) casos foram estágio 2, e 1 (1,3%) paciente foi estágio 3a. Eventualmente, quando a DRC foi descrita como TFG <90 mL/min/1,73 m^2^, a frequência da DRC foi de 9,4% de acordo com a fórmula de Schwartz (à beira do leito), 76,6% segundo a fórmula de TFGe-CisC, e 25,9% segundo a fórmula de TFGe-CrS-CisC em nosso estudo (Tabela 3).

**Tabela 3:** Prevalência de DRC de acordo com as fórmulas de TFG de Schwartz (à beira do leito), TFGe-CisC, e TFGe-CrS-CisC

Estágio	Estágio 1 ≥90 n (%)	Estágio 2 60-89 n (%)	Estágio 3a 45-59 n (%)	Total
TFGe (mL/min/1,73 m^2^)				
Schwartz (à beira do leito)	85 (90,4%)	9 (9,4%)	-	94
TFGe-CisC	18 (23,3%)	53 (68,8%)	6 (7,7%)	77
TFGe-CrS-CisC	57 (74%)	19 (24,7%)	1 (1,3%)	77

Não houve correlação estatisticamente significativa entre DRC e fatores clínicos, tais como DECH aguda e crônica, regime de preparação, ICT, DVO, uso de ICN, cistite hemorrágica associada ao vírus BK, ITU, compatibilidade do doador, desenvolvimento de sepse, internação em UTI e medicação antimicrobiana nefrotóxica de acordo com as equações de Schwartz (à beira do leito), TFGe-CisC e TFGe-CrS-CisC (p>0,05). Não houve relação significativa entre LRA e reativação do CMV e as fórmulas de Schwartz (à beira do leito) e TFGe-CrS-CisC (p=0,907 e p=0,325, respectivamente). Segundo a TFGe-CisC, a reativação do CMV causou um aumento de 4,2 vezes no desenvolvimento da DRC (OR=4,2; p=0,041). Quando calculado com a fórmula de TFGe-CisC, o risco de DRC foi maior em casos que foram submetidos ao TCTH com mais de 120 meses de idade (OR=3,359; p=0,03) (Tabela 4).

**Tabela 4:** Análise de regressão logística univariada de fatores de risco de DRC (TFGe-CisC)

Características (n)	OR (IC 95%)	Valor de p
Idade no TCTH	3,359 (1,127-10,018)	0,03
Doenças malignas	1,070 (0,362-3,166)	0,902
Lesão renal aguda	0,159 (0,033-0,753)	0,02
DECH aguda	0,178 (0,017-1,862)	0,150
DECH Crônica	0,500(0,033-7,541)	0,617
Irradiação corporal total	2,450 (0,743-8,076)	0,141
Doença veno-oclusiva	1,111 (0,266-4,635)	0,885
Inibidor de calcineurina em meses	0,347(0,110-1,098)	0,072
Vírus BK	0,230 (0,028-1,908)	0,174
Internação em terapia intensiva	1,486(0,347-6,458)	0,597
Desenvolvimento de sepse	1,661(0,523-5,280)	0,390
Reativação do CMV	4,2 (1,059-16,259)	0,041
Uso de ganciclovir	0,422 (0,140-1,275)	0,126
Uso de vancomicina	1,247(0,343-4,529)	0,738
Uso de anfotericina B	1,587(0,425-5,933)	0,492
Uso de amicacina	2,763 (0,717-10,651)	0,140

Itens com significância estatística (P<,05) são mostrados em negrito. DECH: doença do enxerto contra hospedeiro, CMV: citomegalovírus.

Com relação à proteinúria, hematúria e hipertensão por insuficiência renal a longo prazo, a proteína/creatinina na amostra isolada de urina foi >0,21 g/dia em 9 (9,4%) casos, e microalbuminúria foi encontrada em 18 (18,8%) pacientes. Constatou-se que a β2- microglobulina, examinada em termos de funções tubulares, foi >300 mcg/L em 9 (9,4%) casos. Hematúria foi observada em 13 (13,5%) casos. Sessenta e oito (72,3%) casos eram normotensos, 22 (23,4%) casos eram pré-hipertensos, e 4 (4,3%) casos eram hipertensos.

## Discussão

Embora o TCTH-alo seja utilizado com frequência crescente no tratamento de muitas doenças, as preocupações com complicações a longo prazo permanecem. Uma das complicações a longo prazo do TCTH-alo é a DRC. O desenvolvimento de DRC em receptores de TCTH-alo tem importantes consequências médicas e econômicas, como DRET e morbidade cardiovascular, e pode diminuir a qualidade de vida. Embora as complicações renais iniciais após o TCTH sejam bem definidas, os efeitos a longo prazo ainda são menos conhecidos^13,14^.

Neste estudo, diferentemente de outros estudos, as funções renais de longo prazo e os fatores de risco para doença renal foram avaliados com diferentes fórmulas de TFGe usando CisC sérica e CrS em pacientes sem recidiva pelo menos 2 anos após receberem o TCTH-alo. A TFGe foi avaliada de acordo com as fórmulas de Schwartz (à beira do leito), TFGe-CisC, e TFGe-CrS-CisC. Como a CrS é afetada pela desnutrição ou secreção tubular, neste estudo calculamos a TFGe com equações baseadas em CisC além da CrS. Quimioterápicos e a radioterapia utilizados no regime preparatório, drogas antimicrobianas nefrotóxicas, LRA e DECH têm demonstrado estar entre os fatores de risco de DRC após o TCTH^15^. No estudo de Abboud et al.^16^, foi encontrada disfunção renal em 7% das crianças com TCTH, e o desenvolvimento de DRC foi associado à ICT e à DECH crônica. A administração de ICT durante a preparação e a DECH crônica é uma das principais causas de doença renal tardia^17^. Além disso, foi demonstrado que a idade de transplante superior a 84 meses é um risco para baixa TFGe a longo prazo após TCTH em todos os pacientes^18^. No estudo realizado por Sakellari et al.^19^, a incidência de DRC foi de 20,4%. Além disso, DECH crônica, transplante de doador não relacionado, LRA pós-transplante e idade avançada foram identificados como fatores de risco para DRC^19^. Em nosso estudo, em casos avaliados como DRC de acordo com as três fórmulas, o desenvolvimento da DRC não foi associado a ICT, vírus BK, sepse, DVO, tipo de doador e DECH crônica. Entretanto, em pacientes identificados como DRC segundo a equação TFGe-CisC, verificou-se que idade de transplante acima de 120 meses, infecção por CMV e LRA estavam associados à DRC. Nosso estudo enfatiza a importância da CisC em casos de TCTH, uma vez que os fatores de risco estão associados somente à DRC identificada pela fórmula TFGe-CisC. Muto et al.^20^ revelaram a relação entre CisC e o uso de ICN. Uma forte correlação inversa também foi observada entre CisC e TFGe. Tudo isso sustenta que a CisC pode ser um marcador clínico útil para definir o risco de DRC em receptores de TCTH-alo. Quanto mais cedo a DRC for diagnosticada, melhor o progresso para DRET pode ser retardado por meio de tratamento e precauções.

Diferentes resultados foram obtidos em diferentes publicações que investigam a frequência de DRC em casos de TCTH-alo. No estudo de Abboud et al.^16^, a disfunção renal foi encontrada em 7% e no estudo de Hingorani et al.^21^, constatou-se que a prevalência de DRC foi de 23% após o TCTH. Y. Ukeba-Terashita et al.^18^ e Sakellari et al.^19^ relataram incidências de DRC de 21,7% e 20,4%, respectivamente. Em nosso estudo, diferentes taxas de prevalência de DRC foram determinadas com diferentes fórmulas de TFGe. A prevalência de DRC foi de 9,3% de acordo com a equação de Shwartz (à beira do leito), 76,6% de acordo com a equação da TFGe-CisC, e 25% de acordo com a equação da TFGe-CrS-CisC. Isto sugere que a fórmula de Shwartz (à beira do leito), baseada na CrS, que usamos com mais frequência na prática diária, pode nos induzir em erro na determinação de DRC. Por outro lado, a detecção da DRC pode ser atrasada por meio do cálculo da TFGe com base na CrS em casos de TCTH, que é um grupo de pacientes onde o diagnóstico e tratamento precoces de DRC são muito importantes.

O uso de fórmulas baseadas em CrS para estimar a TFG é potencialmente problemático. A creatinina é excretada através da filtração glomerular e da secreção tubular. A concentração de CrS pode permanecer normal mesmo que a TFG tenha caído para 50% do intervalo normal. Por outro lado, a CisC é expressa por todas as células e é excretada somente através da filtração glomerular. Além disso, essa substância não é secretada, mas sim reabsorvida e catabolizada por células epiteliais tubulares, impedindo assim o retorno ao sangue. A CisC oferece sensibilidade diagnóstica superior na detecção da TFG ligeiramente comprometida em comparação com a CrS^21^. Hazar et al.^23^ relataram recentemente que a TFG baseada em CisC pode estar muito mais próxima do método de TFG com base em 99mTc-DTPA atualmente aceito. Neste estudo, foi encontrada uma forte correlação entre o nível de CisC e a TFGe. O nível sérico de CisC é, portanto, útil na predição da função renal em pacientes com TCTH. Além disso, a CisC pode ser medida sem coleta de urina, proporcionando uma grande vantagem para o fácil monitoramento da função renal em ambientes pré-clínicos^24^.

A CrS é o marcador mais comumente utilizado para a avaliação da função renal. A CisC foi proposta como um marcador alternativo de função renal para a TFGe, que parece ser mais precisa do que a CrS. A TFGe é considerada o melhor índice geral de função renal, permitindo o reconhecimento precoce de disfunção renal, bem como a dosagem precisa de drogas imunossupressoras. Deve-se enfatizar a forte dependência da CrS com relação à idade, sexo, estado nutricional e massa muscular magra. Esta limitação é particularmente importante em pacientes nos quais a CrS pode ser enganosamente baixa devido à perda muscular ou hiperfiltração. Assim, a CrS só pode ser utilizada como um indicador bruto de função renal significativamente prejudicada, não é um marcador ideal para o reconhecimento precoce de lesão renal aguda, e também não reflete alterações de TFGe em tempo real. Portanto, em receptores de TCTH-alo, as equações baseadas em creatinina têm resultados equivocados na estimativa da TFG. A CisC é um marcador de função renal que apresenta melhor sensibilidade diagnóstica do que a CrS para a detecção de deficiências leves a moderadas da TFG. Tem sido relatada como sendo menos afetada pela idade, sexo e composição corporal^25^. Estudos recentes demonstraram que a fórmula baseada em CisC teve um melhor desempenho preditivo para TFGe do que a fórmula baseada em CrS.

Neste estudo, observou-se que o risco de DRC foi bastante elevado em crianças que foram submetidas ao TCTH-alo por diferentes razões (TFGe-CisC de 76,6%). Semelhante à literatura, encontramos infecção por CMV, idade de transplante superior a 120 meses e LRA prévia como fatores de risco para DRC. Outro achado importante de nosso estudo foi que a frequência de DRC variou dependendo das fórmulas de TFGe. Como a DRC é frequentemente assintomática nos estágios iniciais, a detecção precoce é importante para prevenir ou retardar a progressão para DRET. Neste sentido, a fórmula da TFGe-CisC pode ser mais eficaz do que a da CrS na definição de DRC após TCTH-alo. A CisC proporcionou melhores resultados entre as fórmulas de TFGe pediátricas em pacientes com TCTH-alo. A DRC-TFGe-CrS-CisC é atualmente considerada a melhor equação preditiva de TFG para avaliação da progressão da DRC na faixa etária pediátrica. A nova equação FAS (*full age spectrum*) é baseada na creatinina sérica normalizada (CrS/Q), onde Q é a CrS mediana de populações saudáveis considerando a idade e o sexo^26^. Não utilizamos a fórmula FAS, pois nosso estudo consistiu apenas em pacientes pediátricos.

As limitações de nosso estudo incluem o desenho transversal, a falta de relação entre a TFGe basal e a DRC, a não homogeneidade dos grupos de pacientes, períodos de acompanhamento, regimes preparatórios, idade no momento do transplante, tipo de doador e diagnósticos primários.

Em conclusão, a medição de CisC é superior à medição de CrS para a avaliação de disfunção renal e pode fornecer uma ferramenta útil para identificar o risco de DRC em receptores de TCTH-alo. A TFGe-CisC pode ser considerada o método de avaliação de TFGe mais adequado em transplantes pediátricos.
