# Supplementary material for: Erratum: Long-term kidney outcomes in children after allogeneic hematopoietic stem cell transplantation assessed with estimated glomerular filtration rate equations, creatinine levels, and cystatin C levels
Source: J Bras Nefrol. 2025 Apr 25;47(2):e20210231er. doi: 10.1590/2175-8239-JBN-2021-0231eren (PMC12023911; doi:10.1590/2175-8239-JBN-2021-0231eren)
Supplement: Translation

# Translation: Errata: Desfechos renais a longo prazo em crianças após transplante alogênico de células-tronco hematopoiéticas avaliados com equações de taxa de filtração glomerular estimada, níveis de creatinina e níveis de cistatina C

No artigo “Desfechos renais a longo prazo em crianças após transplante alogênico de células-tronco hematopoiéticas avaliados com equações de taxa de filtração glomerular estimada, níveis de creatinina e níveis de cistatina C”, com número de DOI: https://doi.org/10.1590/2175-8239-JBN-2021-0231pt, publicado no periódico Brazilian Journal of Nephrology, 45(1):60–66, 2023, na página 66 estava faltando os agradecimentos:
